# Germany Outbreak of Escherichia Coli, Lessons to Be Learned

**Published:** 2011-06-01

**Authors:** K B Lankarani

**Affiliations:** 1 Health Policy Research Center, Shiraz University of Medical Sciences, Shiraz, Iran

One of the most severe outbreaks of Escherichia coli has recently occurred in Europe.[1] The epidemic was started in Germany but now has affected more than 16 countries with more than 3200 reported diseased and 35 death cases.[2][3] Of the reported 812 cases with hemolytic uremic syndrome (HUS), 23 died with a case fatality of 2.8%. Death toll among 2443 infected patients with Enterohemorrhagic E. coli (EHEC) infection not complicated with HUS was 12, indicating a case fatality rate of nearly 0.5%.[2] Most cases were from Germany, but 15 other countries including 13 European member states and USA and Canada also reported occurrence of the disease.[2] The second country with the highest number of reported cases was Sweden followed by Denmark.[2] Globally, all but 5 cases occurred among people who had recently traveled to Germany or were living there. Except one Swedish patient who died with HUS, all other 34 reported deaths were from Germany including 22 patients with HUS and 12 others with EHEC infection without HUS. Most cases were female and the highest attack rate was in age range of 20-49 years and less than 15% of cases aged less than 20 years old. These figures are different from the previous epidemics of HUS and EHEC which had a higher attack rate in youngsters.[2][4]

The causative organism was found to be enteroaggregative verocytotoxin-producing E. coli (EAggEC VTEC) O104:H4 bacterium.[2][5] This strain is quite unusual and it seems to be an hybrid strain between a Shiga-toxin-producing E. coli (STEC) strain and an enteroaggregative E. coli strain.[5]

HUS is usually complicated in 6-9% of infections with O157:H7 EHEC, the most common strain of EHEC reported previously, but in this epidemic, the reported incidence was nearly 25%.[2][4] Although a referral bias is possible, but it is quite clear that the occurrence of HUS is much higher with this unusual strain. For 0157:H7 strain, the most common vehicle for transmission is undercooked beef specially the ground beef.[4] This O104:H4 strain was found to be transmitted by bean and seed sprouts as was reported by German authorities in June 10th.[2] Overall, it seems that we are now confronted with a more aggressive organism with somehow different microbiological and clinical characteristics. Detailed studies are underway and probably we would hear more on differences of this emergent strain with its predecessor strains.[3]

The most challenging question right now is why this epidemic was reported so late to international health authorities.According to German Federal Agency for Disease Surveillance, the first cases were hospitalized in Germany in May 1st.[3] But it was only 3 weeks later when the possibility of an epidemics of E. coli was reported to European Center for Disease Prevention and Control.[3] Although the unusual nature of this strain both in microbiologic characteristics and clinical presentations as discussed above was blamed for this unacceptable delay, the real problems should be sought elsewhere. Absence of an active national authority for disease control is probably one of the weak points as was mentioned by some German scientists.[3]

The dissociation between health systems and disease surveillance organizations which is present in many countries is a persistent defect which makes repetition of this sad event quite possible everywhere and at anytime. When there is no systematic connection between providers of medical care and the surveillance organizations and when there is no clear definition of the level of hazards which makes decisions mostly personal not evidence based or targeted, it would not be rare to see this amount of delay in reporting.

According to International Health Regulations (IHR) approved by World Health Assembly, all member states of World Health Organization (WHO) have to strengthen their existing public health surveillance and response.[6] The main purpose of IHR is to help the international community to prevent and respond to acute public-health risks that have the potential to cross borders and threaten people worldwide. IHR entered to force in June 15th, 2007. Now on the 4th anniversary of this important event in collective global health action, it is a good time to reemphasize the importance of strengthening the national health surveillance systems.

A pyramidal model of integrated organized and comprehensive approach which is flexible, continuous and dynamic which makes possible to have a collective action of all players from community, government and international agencies is a good model for effective surveillance and response.[7] Through this model, better inter and intra-sectoral collaboration in surveillance and response can become a reality.

In countries with a national primary health care system like public health network in Iran, the effectiveness of this primary health care based system of surveillance has been shown in control of both communicable as well as non-communicable diseases.[7][8] We do not want to neglect the importance of molecular techniques in the surveillance of microbial diseases like the current EHEC epidemics but if the starting point for a surveillance system becomes the primary health care system, then we would see more rapid detection of outbreaks and more rapid responses. This also could get a better support from the community as well as the non-governmental organizations.
